# Hematemesis on an esophageal foreign body revealing a double aortic arch: a case study

**DOI:** 10.1093/jscr/rjae057

**Published:** 2024-02-13

**Authors:** Winga Foma, Damessane Lamboni, Gérémie Ananidjin, Uziel Boko, Dodji Koulekey, Sosso Piham Kebalo, Lantam Sonhaye, Bathokédéou Amana, Essohanam Boko

**Affiliations:** ENT Department, Sylvanus Olympio University Hospital, Lomé 99345, Togo; Surgical Emergency Department, Sylvanus Olympio University Hospital, Lomé 99345, Togo; ENT Department, Sylvanus Olympio University Hospital, Lomé 99345, Togo; ENT Department, Sylvanus Olympio University Hospital, Lomé 99345, Togo; Surgical Emergency Department, Sylvanus Olympio University Hospital, Lomé 99345, Togo; Pediatric Surgery Department, Sylvanus Olympio University Hospital, Lomé 99345, Togo; Radiology and Medical Imaging Department, Campus University Hospital, Lomé 99345, Togo; ENT Department, Sylvanus Olympio University Hospital, Lomé 99345, Togo; ENT Department, Campus University Hospital, Lomé 99345, Togo

**Keywords:** esophageal foreign body, double aortic arch, dysphagia lusoria, haematemesis

## Abstract

A double aortic arch is a rare abnormality of the aortic arch caused by the persistence of the distal part of the right dorsal aorta. It can be manifested by respiratory and/or digestive symptoms. We report a case of double aortic arch revealed by an esophageal foreign body complicated by haematemesis in a 13-year-old boy having required multidisciplinary care.

## Introduction

A double aortic arch is a rare anomaly of the aortic arch due to the persistence of the distal part of the right dorsal aorta [1]. It may be revealed early in the neonatal period by respiratory disorders, or much later in adults by digestive disorders [2]. Thoracic angio-computed tomography (angio-CT) scan is the gold standard for diagnosis. Treatment is surgical but indicated in cases of severe disorders [3]. We report the case of a 13-year-old patient, treated for an oesophageal foreign body (OFB) complicated by haematemesis revealing a double aortic arch. This case also illustrates a rare etiological diagnosis of dysphagia lusoria.

## Observation

**Figure 1:**
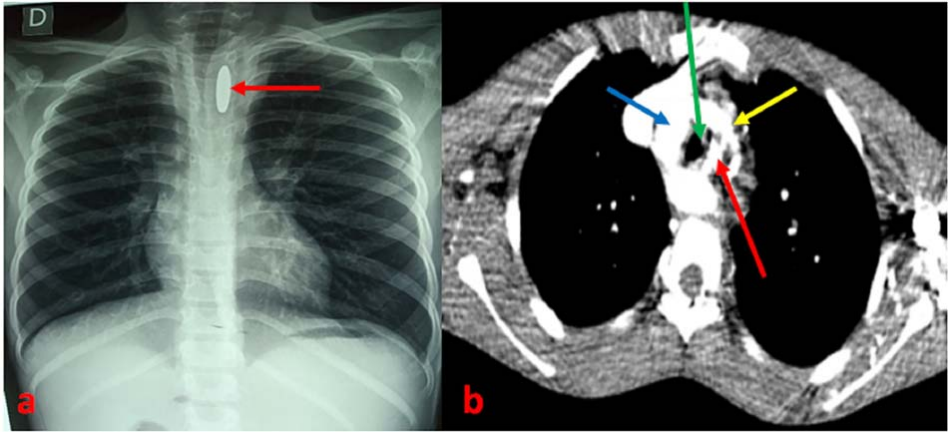
(a) Chest X-ray showing an opacity related to a metallic foreign body in an enlarged mediastinum with narrowing of the thoracic trachea. (b) CT scan section showing a metallic foreign body embedded in the esophagus associated with a double aortic arch. Arrows: red (esophageal foreign body), blue (right arch), green (trachea), and yellow (left arch).

A 13-year-old patient was referred from a peripheral health center to the ENT department of the CHU Sylvanus Olympio for better management of hematemesis due to an esophageal foreign body. He presented with dysphagia to solids and progressive weight loss secondary to accidental ingestion of a coin 6 months previously, which he had not reported to his parents. With the onset of a chronic cough without sputum and moderate hematemesis, he was taken to a peripheral health center, where a chest X-ray revealed a metallic opacity with a large longitudinal axis in the enlarged upper mediastinum and a narrowing of the thoracic trachea (Fig. 1a). In his history, the parents reported a notion of undocumented intermittent dysphagia to solids, yielding spontaneously, evolving since early childhood but worsening in the last few months. On admission, the child’s temperature was normal, he had delayed weight and height, his general condition was preserved, and his hemodynamic status was stable. Examination of the cardiovascular, pleuropulmonary, and digestive systems was unremarkable. A few hours after admission, the patient presented with profuse hematemesis and hemorrhagic shock, which was compensated by transfusions of packed red blood cells. A thoracic CT scan with contrast injection was ordered to understand the mechanism of blockage of the foreign body, to assess its relationship with neighboring organs, and to understand the occurrence of haematemesis. This revealed that a round metallic foreign body, ~2.5 cm in diameter, embedded in the hypertrophied oesophageal muscle, at the level of the 3rd and 4th thoracic vertebrae, in a double aortic arch oesophageal stenosis with a right arch predominantly retro-oesophageal (Fig. 1b).

**Figure 2:**
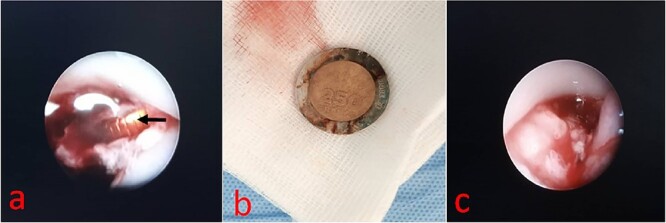
(a) Endoscopic view of esophageal foreign body. (b) 250FCFA coin. (c) Endoscopic view of the posterior esophageal wall.

The diagnosis of a neglected esophageal foreign body complicated by hematemesis on a double aortic arch was made. Rigid-tube esophagoscopy under general anesthesia, with armed surgical expectation, was indicated. Intraoperatively, we discovered a metallic foreign body located 18 cm from the upper dental arch engulfed in a cluster of clots and granulomatous tissue (Fig. 2a). A 250 CFA francs coin was extracted by gentle traction with forceps (Fig. 2b). The posterior wall of the esophagus was inflamed and remodeled, with no active bleeding (Fig. 2c).

**Figure 3:**
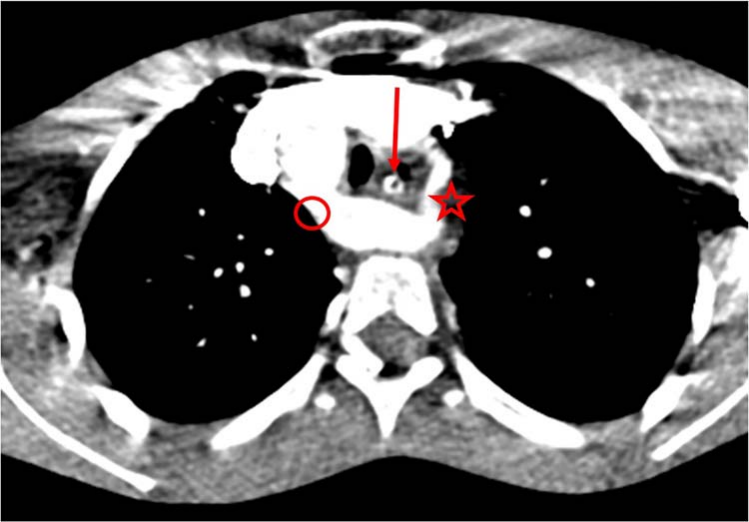
Double aortic arch on thoracic angio-CT scan: arrow (esophagus), star (left arch), circle (right arch).

Post-operative medical treatment consisted of a nasogastric feeding tube, parenteral administration of omeprazole (40 mg/day), and amoxicillin/clavulanic acid (02 g/day in two doses) for 10 days. On postoperative Day 6, the patient presented with profuse haematemesis, requiring macromolecular infusion and transfusion of packed red blood cells. A thoracic angio-CT scan was performed the following day and revealed the same lesions as on the CT scan, with no images suggestive of aneurysms or oesovascular fistulas (Fig. 3). Omeprazole (20 mg/day) per os was continued for 6 weeks, and meal splitting was advised. The patient was subsequently lost to follow-up, but telephone contact revealed that there had been no recurrence of bleeding at 2 years’ follow-up.

## Discussion

The double aortic arch is due to the persistence of the distal part of the right dorsal aorta, resulting in the formation of right and left aortas that surround the trachea and esophagus before joining to form the descending thoracic aorta. Each arch provides the origin of the primitive carotid artery and the homolateral subclavian artery. Usually, both aortic arches are patent, in which case the right or left may be dominant, or they may be of equal size. In the literature, the right side is dominant in 75% of cases, as was the case in our patient [1]. The double aortic arch is one of the embryological variations of the aortic arches which are most often unrecognized and/or asymptomatic, but which can be complicated by tracheoesophageal symptoms due to extrinsic compression [2]. Symptoms of the duplication may present from infancy to childhood, depending on the severity of oesotracheal compression and the existence of associated malformations. According to the authors, dysphagia to solids is more frequently observed in adults and older children, as was the case in the present observation [3–8]. In infants, symptoms are dominated by stridor and respiratory distress [3]. Chronic dysphagia to solids related to esophageal compression by a vascular ring or aberrant retro esophageal right subclavian artery is known as dysphagia lusoria and was first described in 1794 by David Bayford [4, 5]. This anomaly increases the physiological narrowing of the interaortic-caval portion of the thoracic esophagus. When dysphagia is associated with the enlargement of the mediastinum, tracheal narrowing or malposition, or absence of the aortic knob on the thoracic X-ray, an encircling anomaly of the aortic arch should be strongly suspected, with diagnostic certainty based on thoracic angio-CT scan [2]. Furthermore, in the presence of a radio-opaque OFB with an atypical position of the OFB (with a long longitudinal axis in an enlarged mediastinum, as in the present case), an encircling anomaly should be evoked. In our patient, extrinsic esophageal compression would have prevented the migration of the coin and favored the aggravation of pre-existing dysphagia. Complications such as haematemesis can occur in cases of oesophageal perforation or oesovascular fistula and are often due to sharp foreign bodies [8]. In our case, the hematemesis was due to the erosion of the posterior wall of the esophagus, with the foreign body embedded in the esophageal muscle. However, a case of spontaneous hematemesis without a foreign body, due to fistulization of a Kommerell diverticulum, another variant of aortic arch anomalies, has been reported in an elderly subject [6].

Double aortic arches are treated surgically. It consists of a ligation-section of one arch with release of the esophagus and trachea via thoracotomy, or selective embolization via endoscopy [9, 10]. Nevertheless, the indication for surgery need not be systematic, but it is definite if esotracheal compression syndrome is severe [1]. In the case reported, given the regression of symptoms after extraction of the coin and the favorable evolution over the weeks, we indicated a fractioning of meals and monitoring.

## Conclusion

The double aortic arch is a rare pathology. It may be discovered during morphological investigations of dysphagia. It should be considered when a foreign body is embedded in the upper mediastinum. Angio-CT scan remains the most sensitive diagnostic tool. Surgical treatment is indicated if symptoms are obvious and/or life-threatening.
